# A Novel Assay in Whole Blood Demonstrates Restoration of Mitochondrial Activity in Phagocytes After Successful HSCT in Hyperinflamed X-Linked Chronic Granulomatous Disease

**DOI:** 10.1007/s10875-022-01338-x

**Published:** 2022-08-10

**Authors:** Maddalena Migliavacca, Luca Basso Ricci, Giada Farinelli, Valeria Calbi, Francesca Tucci, Federica Barzaghi, Francesca Ferrua, Maria Pia Cicalese, Silvia Darin, Lina Raffaella Barzaghi, Fabio Giglio, Jacopo Peccatori, Francesca Fumagalli, Roberto Nicoletti, Stefania Giannelli, Claudia Sartirana, Alessandro Bandiera, Maria Esposito, Raffaella Milani, Benedetta Mazzi, Andrea Finocchi, Sarah Marktel, Andrea Assanelli, Franco Locatelli, Fabio Ciceri, Alessandro Aiuti, Maria Ester Bernardo

**Affiliations:** 1 San Raffaele Telethon Institute for Gene Therapy (SR-Tiget), IRCCS San Raffaele Scientific Institute, Pediatric Immunohematology and Bone Marrow Transplantation Unit, San Raffaele Scientific Institute, Via Olgettina, 60, 20123 Milan, Italy; 2 Paediatric Immunohematology and Bone Marrow Transplantation Unit, IRCCS San Raffaele Scientific Institute, Milan, Italy; 3 Fondazione Telethon, Milan, Italy; 4 Neurosurgery department, IRCCS San Raffaele Scientific Institute, Milan, Italy; 5 Hematology and Bone Marrow Transplantation Unit, IRCCS San Raffaele Scientific Institute, Milan, Italy; 6 Department of Radiology, IRCCS San Raffaele Scientific Institute, Milan, Italy; 7 Thoracic surgery, IRCCS San Raffaele Scientific Institute, Milan, Italy; 8 S.O.C. Malattie Infettive ASL VC Piemonte, Milan, Italy; 9 Cytometry Laboratory, IRCCS San Raffaele Scientific Institute, Milan, Italy; 10 HLA Laboratory, IRCCS San Raffaele Scientific Institute, Milan, Italy; 11 Department of Paediatrics, Ospedale Pediatrico Bambino Gesù and University of Rome “Tor Vergata”, Rome, Italy; 12 Department of Onco-Haematology and Cell and Gene Therapy, IRCCS Bambino Gesù Children’s Hospital, Rome, Italy; 13 Department of Pediatrics, Sapienza University of Rome, Rome, Italy; 14 Università Vita-Salute San Raffaele, Milan, Italy

**Keywords:** Primary Immunodeficiency, Phagocytes disorders, X linked chronic granulomatous disease, Hematopoietic stem cell transplantation, Mitochondrial activity, X linked chronic granulomatous disease carriers, Novel protocol on whole blood, strong direct correlation between mitochondrial activity, hematopoietic stem cell transplantation chimerism and DHR monitored before and after transplantation and in XCGD carriers

## Abstract

X-linked chronic granulomatous disease is a rare disease caused by mutations in the CYBB gene. While more extensive knowledge is available on genetics, pathogenesis, and possible therapeutic options, mitochondrial activity and its implications on patient monitoring are still not well-characterized. We have developed a novel protocol to study mitochondrial activity on whole blood of XCGD patients before and after transplantation, as well as on XCGD carriers. Here we present results of these analyses and of the restoration of mitochondrial activity in hyperinflamed X-linked Chronic Granulomatous Disease after hematopoietic stem cell transplantation. Moreover, we show a strong direct correlation between mitochondrial activity, chimerism, and DHR monitored before and after transplantation and in XCGD carriers. In conclusion, based on these findings, we suggest testing this new ready-to-use marker to better characterize patients before and after treatment and to investigate disease expression in carriers.

## Introduction

X-linked chronic granulomatous disease (XCGD) is a rare, genetic disease characterized by inactive NADPH oxidase complex leading to a predisposition towards severe life-threatening infections, hyperinflammation, and immune dysregulation. Hematopoietic stem cell transplantation (HSCT) is the only curative option with an excellent outcome, as recently described in a large cohort [1]. However, older patients, who carry a substantial disease burden, and recipients of antigen mismatched grafts have a less favorable outcome. Recently, ex vivo gene therapy with hematopoietic stem cells showed promising preliminary results [2].

Reactive oxidative species (ROS) are known to be reduced in XCGD phagocytes; however, mitochondrial ROS (mtROS) levels are still not well-characterized. Stimulated mtROS are reduced in granulocytes of XCGD patients [3–6] while basal XCGD mtROS were reported to be increased as compared to healthy donors [7]. MtROS levels have not been investigated in carriers or after HSCT.

MitoSOX™ Red superoxide indicator is a fluorogenic dye specifically targeted to mitochondria in live cells usually tested by flow-cytometry on peripheral blood (PB) mononuclear cells. However, cell separation leads to reduced cell viability and possible loss of in vitro reproducibly of mitochondrial function. For this reason, we set a novel protocol to test MitoSOX™ on patients’ whole blood followed by the gating of phagocytes to be more reproducible, standardizable, and time-saving.

## Methods

PB after red blood cells lysis was labeled with fluorescent antibodies against CD14 BV510 (BD Biosciences) + CD 15 (APC) (Biolegend). We added 5μM MitoSOX™ for 10 min at 37°C. We divided the samples into two parts and added in stimulated samples 0.5 μl PMA 1.62 mM for 30 min at 37°C. All samples were acquired through BD FACSCanto™ in 30 min after Rainbow beads (Spherotech) calibration and raw data were collected using the DIVA software (BD Biosciences). The data were subsequently analyzed with the FlowJo software Version 9.3.2 (TreeStar) and the graphical output was automatically generated through Prism 6.0c (GraphPad software).

**Fig. 1.**
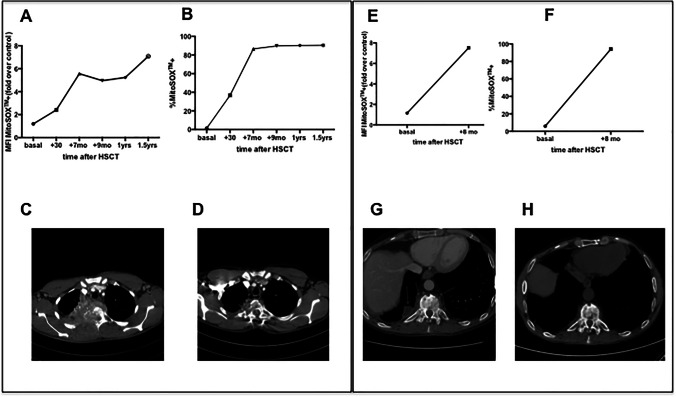
MitoSOX^+^ MFI (**A**) (**E**), percentage (**B**) (**F**) on granulocytes on whole blood and chest CT before (**C**) (**G**) and after HSCT (**D**) (**H**) in PT1 and in PT2 respectively

Statistical analyses were performed with Prism 6.0c (GraphPad software). Analytical tests for statistical significance among groups used the Mann-Whitney test (*P* values are specified in each figure) with the Bonferroni correction. The Spearman’s rank correlation coefficient was considered for studies of correlation (Fig. 1).

**Fig. 2:**
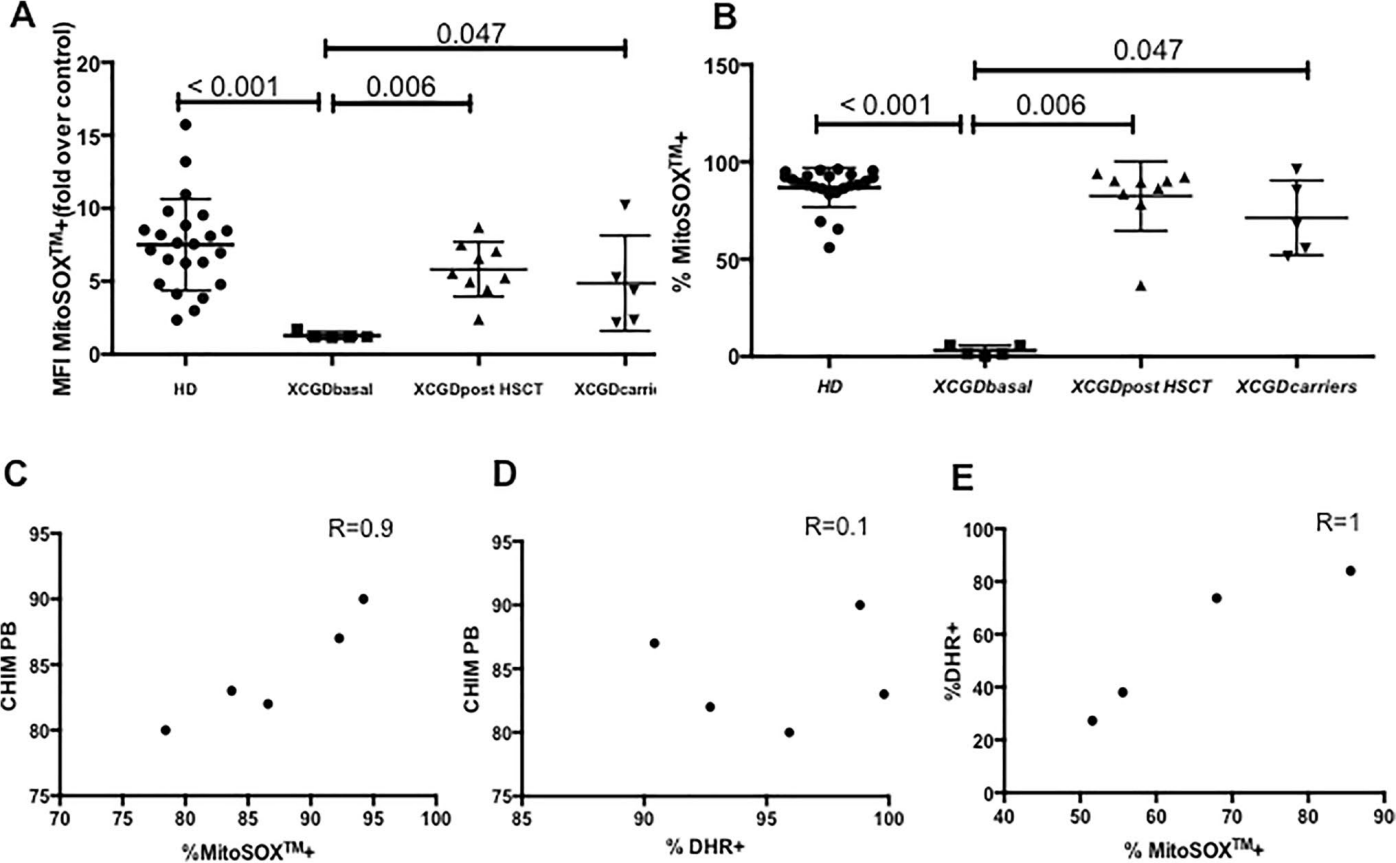
MitoSOX^+^ MFI (**A**) and percentage (**B**) on granulocytes on whole blood; correlation of chimerism/ mitoSOX post HSCT (**C**), chimerism/DHR post HSCT (**D**), mitoSOX/DHR in carriers (**E**)

Hematopoietic chimerism was analyzed using quantitative Real Time-PCR. Briefly, host and/or donor polymorphisms for subsequent chimerism monitoring were selected using AlleleSEQR chimerism assay, in which there are 34 probes available for insertion-deletion polymorphisms spread over 19 different chromosomes by TaqMan qPCR reactions. For each donor-recipient pair, two markers for the patient and/or for the donor were selected. The ∆∆Ct method or comparative CT method for relative quantitation was used to determine the percentage according to the formula 2-∆∆CT×100. The averages between two independent markers analyzed were calculated for each time-point for patient and/or donor. Chimerism was performed on CD15+ cells isolated from peripheral blood whenever enough material was available; in two samples it was performed on whole blood (corresponding to the data points > 87%, see Fig. 2).

**Fig. 3:**
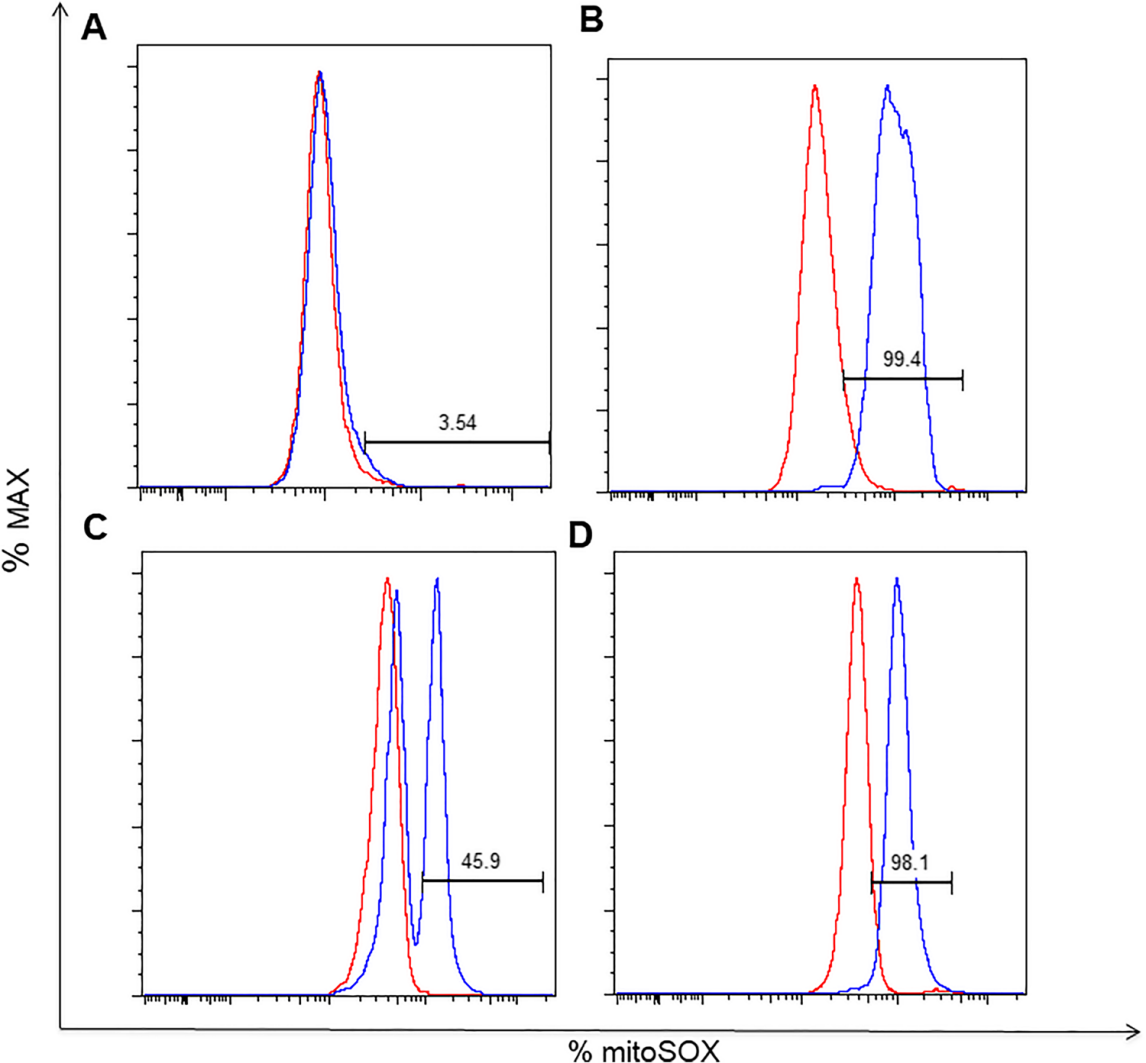
Representative histograms for granulocyte mitoSOX+. In red before PMA stimulation, in blue after PMA stimulation. In PT1 basal (**A**), in PT1 at 1.5 years after HSCT (**B**), in a carrier (mother of PT2) (**C**), and in a healthy donor on the same day (**D**)

Dihydrorhodamine (DHR) analysis was performed on granulocytes by Phagoburts (BDBiosciences, Milan, Italy) according to manufacturer’s instructions and analyzed by flow-cytometry in 30 min. Fluorescence was measured before and after stimulation with the protein kinase C activator, phorbol 12-myristate 13-acetate (PMA). The red line represents the unstimulated condition; the blue line indicates the PMA-stimulated condition (see Fig. 3).

**Fig. 4:**
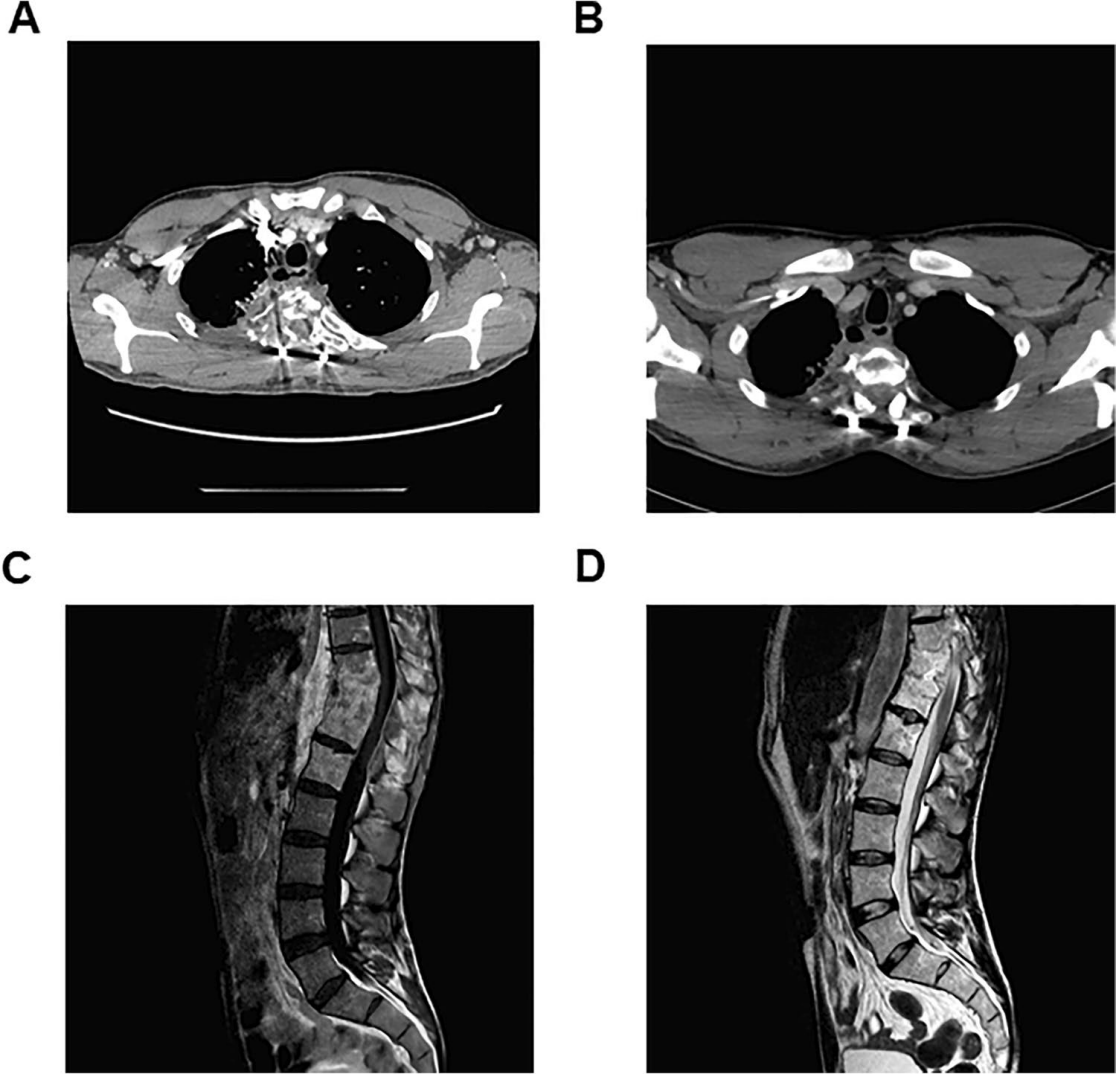
**A** PT1 CT chest: vascularized pathological tissue infiltrating lung, pleura, chest wall (rib and dorsal vertebrae), esophageal fistula. **B** PT1 CT chest: clear reduction of pathological tissue devoid of vascularization with spinal stabilization. Partial resolution of esophageal fistula. **C** PT2 MRI dorso-lumbar spine: spondylodiscitis (vertebral edema and multiple paravertebral fluid collections). **D** PT2 MRI dorso-lumbar spine: complete resolution of vertebral edema and fluid paravertebral collections

Peripheral blood samples were collected after obtaining informed consent according to a protocol approved by San Raffaele Ethical Committee (Fig. 4).

## Combined Results and Discussion

Here we report basal mtROS levels and restoration of stimulated mitochondrial activity in phagocytes of two adult XCGD patients who underwent successful HSCT; results were then confirmed in other XCGD subjects and carriers.

The first two patients presented typical features of the disease with severe, life-threatening infections and were initially managed conservatively. They were referred to our center as young adults due to worsening of infectious episodes and we offered them HSCT.

Patient 1 (PT1, mutation CYBB c.535G>A p.Gly179Arg) was admitted with a right apical pulmonary mass with mediastinal and thoracic wall invasion at the age of 23 years (Fig. 1). PCR on lung biopsy resulted positive for a fungal infection and the patient was started on voriconazole. After 2 months, he developed neurological signs with sensory disorders in the dorsal region, weakness of lower limbs, hyper-reflexia, voiding dysfunction and fever. Chest CT scan showed worsening of the pulmonary lesion, involvement of thoracic vertebrae (Th2, Th3, Th4), and spinal cord compression. He was started on amphotericine-B, wide spectrum antibiotics and dexamethasone. He underwent surgery with arthrodesis, laminectomy, and decompression. Surgical biopsy resulted positive for *Aspergillus clavatusflavus* (by PCR). Resistance to voriconazole (MIC > 32 mg/L) and to amphotericine-B (MIC > 32 mg/L) and sensitivity to posaconazole (MIC 0.05 mg/L) and to itraconazole (MIC 0.5 mg/L) were determined by sensitivity test in vitro. The patient was therefore treated with posaconazole by mouth.

Patient 2 (PT2, mutation CYBB c.676C>T p.R226X) presented recurrent episodes of pneumonia localized in the right inferior lobe, one of these being caused by *Aspergillus fumigatus* and *Mycobacterium chelonae* treated surgically with right lower lobectomy at 20 years of age. Between 20 and 30 years of age, he presented frequent infectious localizations on vertebrae and lungs with granulocytes inflammatory infiltration and epithelial granulomas, accompanied by partial vertebral collapses and cuneization of dorsal vertebrae. Due to back pain and onset of burning dysesthesia in the legs, he underwent CT-guided transpedicular vertebral biopsy with evidence of *Aspergillus Fumigatus* which was sensitive to Voriconazole.

Both patients underwent unrelated donor (9/10 matched) HSCT with PB mobilized hematopoietic stem cells (HSC) after conditioning with Treosulfan, Fludarabine, total body irradiation (2 Gy), and Graft-versus-Host-Disease (GvHD) prophylaxis with post-transplant cyclophosphamide, sirolimus, and mycophenolate mofetil. After HSCT, they showed neutrophil (day +17 and +29) and platelet engraftment (day +14 and +20), respectively, in the absence of acute or chronic GvHD. They experienced an amelioration of ongoing life-threatening infections associated with resolution of the lung and vertebral inflammatory lesions (see Fig. 1). They are alive and in good clinical condition with a significant improvement in their quality of life at 5.9 and 4 years after HSCT, respectively, with donor chimerism >80% in PB.

They were tested for MitoSOX^TM^ on whole blood before and after HSCT. After stimulation with Phorbol Myristate acetate (PMA), PT1 showed a MitoSOX^TM^ expression on phagocytes equal to 1.45% (MFI fold over control 1.19) before HSCT; 36.80% (MFI fold over control 2.40) at 30 days post HSCT, 86.6% (MFI fold over control 5.56) at +7 months, 90.33% (MFI fold over control 5.23) at 1 year and 90.46% (MFI fold over control 7.07) at 1.5 year. PT2 showed a basal of MitoSOX^TM^ in phagocytes of 5.95% (MFI fold over control 1.154) and 94.21 % (MFI fold over control 7.52) at 8 months post HSCT (Fig. 1).

Dihydrorhodamine (DHR) analysis was also performed on granulocytes by Phagoburts^TM^ (BDBiosciences, Milan, Italy) according to manufacturer’s instructions and analyzed by flow-cytometry. MitoSOX^TM^ results paralleled well with DHR positivity after stimulation with PMA and with chimerism analysis on PB in both patients, before and after HSCT. (Fig. 2)

To further support the possible role of MitoSOX staining in monitoring XCGD patients, we analyzed five additional XCGD patients, evaluated before and/or after HSCT, as well as XCGD carriers (*n*=4). A cohort of healthy subjects was used as control (*n*=23). MitoSOX^+^ granulocytes after transplant correlated with chimerism (*R*=0.9) better than DHR positive granulocytes (*R*=0.1).

The percentage of MitoSOX^+^ granulocytes and DHR^+^ granulocytes in carriers suggested a strong direct correlation between these two parameters (*R*=1) (see Fig. 2).

The presence of levels of DHR^+^ granulocytes as high as 85% in carriers has been observed in previous studies [8]. The reason for the different behavior in carrier status vs transplant chimera has to be further investigated. It is true that large cohort studies [1, 9, 10] do not clarify DHR activity in relation to chimerism; on the other hand, one single case report [11] showed a disparity between these two parameters in the post transplant setting.

Overall, these results suggest that measurement of mtROS levels is a useful and ready-to-use marker to monitor granulocyte oxidative function in XCGD patients before and after treatment. MitoSOX™ protocol is an easy and well reproducible assay that can be used in clinical practice with a response available in less than 4 h. Use of rainbow beads can help to standardize and speed up the reading at flow-cytometry. A further advantage of MitoSOX™ is its suitability to be complemented with other markers in larger panels, while DHR emission spectrum is not limited to the FITC channel.

Correlation of mtSOX with DHR in carriers indicates that our test may provide information also on basal residual oxidative activity. An additional role of MitoSOX™ assay can be considered in patients for whom DHR is not informative such as myeloperoxidase deficiency.

Our preliminary analyses suggest mtSOX superiority versus DHR in terms of quantification of the grade of correction after transplant. Mitochondrial activity is an interesting field of study in most genetic diseases. Understanding its modifications after disease treatment could be of help to further elucidate disease mechanisms and to refine the cure of these rare and fatal diseases. Future studies in a larger cohort of subjects will be instrumental to validate the clinical relevance of this novel marker to be employed in the diagnosis and management of XCGD patients.
